# Molecular diversity of rumen bacterial communities from tannin-rich and fiber-rich forage fed domestic Sika deer (*Cervus nippon*) in China

**DOI:** 10.1186/1471-2180-13-151

**Published:** 2013-07-08

**Authors:** Zhi Peng Li, Han Lu Liu, Guang Yu Li, Kun Bao, Kai Ying Wang, Chao Xu, Yi Feng Yang, Fu He Yang, André-Denis G Wright

**Affiliations:** 1 Department of Economical Animal Nutrition and Feed Science, Institute of Special Animal and Plant Sciences, Chinese Academy of Agricultural Sciences, JiLin, China; 2 Department of Animal Science, University of Vermont, 570 Main Street, Burlington, VT 05405-0148, USA

**Keywords:** Ecology, *Prevotella*, Fiber, Tannin

## Abstract

**Background:**

Sika deer (*Cervus nippon*) have different dietary preferences to other ruminants and are tolerant to tannin-rich plants. Because the rumen bacteria in domestic Sika deer have not been comprehensively studied, it is important to investigate its rumen bacterial population in order to understand its gut health and to improve the productivity of domestic Sika deer.

**Results:**

The rumen bacterial diversity in domestic Sika deer (*Cervus nippon*) fed oak leaves- (OL group) and corn stalks-based diets (CS group) were elucidated using 16S rRNA gene libraries and denaturing gradient gel electrophoresis (DGGE). Overall, 239 sequences were examined from the two groups, 139 clones from the OL group were assigned to 57 operational taxonomic units (OTUs) and 100 sequences from the CS group were divided into 50 OTUs. *Prevotella*-like sequences belonging to the phylum Bacteroidetes were the dominant bacteria in both groups (97.2% OL and 77% CS), and sequences related to *Prevotella brevis* were present in both groups. However, *Prevotella shahii*-like, *Prevotella veroralis*-like, *Prevotella albensis*-like, and *Prevotella salivae*-like sequences were abundant in the OL group compared to those in the CS group, while *Succinivibrio dextrinosolvens*-like and *Prevotella ruminicola*-like sequences were prevalent in the CS group. PCR-DGGE showed that bacterial communities clustered with respect to diets and the genus *Prevotella* was the dominant bacteria in the rumen of domestic Sika deer. However, the distribution of genus *Prevotella* from two groups was apparent. In addition, other fibrolytic bacteria, such as *Clostridium populeti* and *Eubacterium cellulosolvens* were found in the rumen of domestic Sika deer.

**Conclusions:**

The rumen of domestic Sika deer harbored unique bacteria which may represent novel species. The bacterial composition appeared to be affected by diet, and sequences related to *Prevotella* spp. may represent new species that may be related to the degradation of fiber biomass or tannins. Moreover, the mechanism and biological functions of *Prevotella* spp. in the rumen ecosystem, and synergistic interactions with other microorganisms should be noticed.

## Background

Sika deer (*Cervus nippon*) represent the most ancient and primitive members of the genus *Cervus* because of the simple structure of their antlers, which is very distinct from those of reindeer. Velvet antlers are one of the main products from Sika deer, and are used in traditional Chinese medicine. In addition, Sika deer yield high quality meat and skin. Domestication of Sika deer began much later than for other ruminants. At present, the number of domesticated Sika deer in China is approximately 550,000 head, most of which are distributed in northwestern China.

In nature, Sika deer graze a wide range of forage types, such as Amur grape, elm, maple, bamboo and some toxic species including Chinese Stellera roots and large flowered larkspurs. Moreover, grazing Sika deer have been observed to prefer tannin-rich plants, such as oak leaves. Similar behavior has also been observed in wild Sika deer (*Cervus nippon yesoensis*) inhabiting the Shiretoko Peninsula of Hokkaido Island in Japan, and in the roe deer (*Capreolus capreolus*) [1,2]. However, domesticated Sika deer held in captivity are commonly fed corn stalks containing a much higher fibrous content. Like other ruminants, Sika deer depend on the rumen for fermentation that involves the conversion of plant fiber to volatile fatty acids. This involves a diverse and dense array of microorganisms, including bacteria, fungi, archaea and protozoa [3]. Among these microorganisms bacterial populations have been extensively studied for many years since rumen bacteria have important roles in the efficient degradation of plant biomass and detoxification of secondary compounds in plants [1,4-7]. This has led to a variety of studies investigating rumen bacterial structure have been conducted on domestic cows, sheep, yak, Reindeer in Norway and wild Sika deer in Japan [4,5,8-10]. Moreover, rumen bacterial communities are affected by the host and diet [11,12]. To our knowledge, very little is known about the rumen bacterial community of domesticated Sika deer in China. A comprehensive understanding of bacterial ecology in the rumen of domesticated Sika deer is necessary to increase the efficiency of fiber digestion and to improve the productivity of velvet antlers.

Thus, we hypotheses the bacterial communities in the rumen of domesticated Sika deer may be unique. And the objectives of the present study were: (1) to describe the bacterial diversity in the rumen from domesticated Sika deer ingesting different diets based on 16S rRNA gene sequence libraries and PCR-DGGE; and (2) to compare the unique rumen bacterial populations of domesticated Sika deer ingesting tannin-rich and fiber-rich materials.

## Results

### Comparative analysis of 16S rRNA gene libraries from two groups

**Figure 1:**
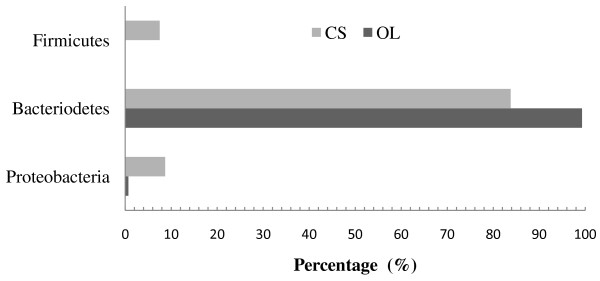
**Composition of 16S rRNA gene libraries at the phylum level. **Clones obtained from the OL and CS groups representing by black and grey bars, respectively.

**Figure 2:**
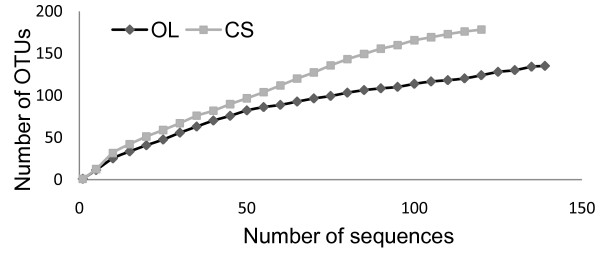
**Rarefaction curves for bacterial 16S rRNA gene libraries. **Dark and gray represent Sika deer feeding on oak leaves-based (OL group) and corn stalks-based (CS group) diets, respectively. Rarefaction curves were generated from the platform MOTHUR using the furthest neighbor method.

A total of 239 non-chimeric sequences were analyzed, 139 sequences from the OL 16S rRNA clone library and 100 sequences from the CS clone library. The two rumen bacterial populations were distinct according to the RDP classifier tool at a confidence threshold of 80% (Figure 1). Within the two groups, members of the phylum Bacteroidetes were the predominant bacteria (99.3% and 85% of clones in the OL and CS groups, respectively). Domesticated Sika deer consuming corn stalks has Firmicutes present whereas they were not found in oak leaves fed domesticated Sika deer. Similarly, Proteobacteria were more expressed in corn stalks than oak leaves diets. The Chao1 (114.2 vs 143.5) and Shannon-Wiener (3.5 vs 3.7) indices of domesticated Sika deer consuming oak leaves were decreased compared to those feeding on corn stalks (Table 1). Moreover, the Libshuff analysis also showed that the bacterial communities between two diets were significantly differed (*P*<0.0001). Rarefaction curves at 3% distance levels revealed 74% and 66% coverage for the OL and CS groups, respectively (Figure 2).

**Table 1:** Number of OTUs, diversity and coverage at 3% distance level using the MOTHUR platform

**Groups**	**Clones**	**OTUs**	**Chao 1**^**a**^	**Shannon-Wiener**^**b**^	**Coverage**
OL	139	57	114.2 (81.1,192.8)	3.5 (3.3,3.7)	0.74
CS	100	50	143.5 (85.8,294.1)	3.7 (3.4,3.8)	0.66

a Chao1 is a nonparametric estimator of the richness in a sample. It is based on the number of rare ribotypes (singletons and doublets) and used to predict the species richness.

b The Shannon-Wiener index is a nonparametric diversity index that combines estimates of richness (total numbers of ribotypes) and evenness (relative abundance of each ribotype) suggesting diversity. It takes into account the abundance of individual taxa and can be used as an overall indicator of the level of diversity in a sample.

Using the software program MOTHUR and a sequence identity criterion cut off of 97%, the 139 OL clone sequences were assigned to 57 OTUs and the 100 CS clone sequences were assigned to 50 OTUs (Table 1).To determine the nearest valid related species, the 16S rRNA gene sequences were compared using GenBank’s Basic Local Alignment Search Tool (BLAST). Within the OL library, 53 of the 57 OTUs (i.e. 97.2% of clones) had 85% or greater sequence identities to genus *Prevotella* (Table 2). Within these OTUs, 23 OTUs (38.1% of clones) showed 87-92% sequence identities to *P. brevis*, 11 OTUs (16.5% of clones) had 86-90% sequence identities to *P. shahii*, 3 OTUs (23.8% of clones) had 91-92% sequence identities to *P. veroralis*, 6 OTUs (12.3% of clones) had distant sequence identities to *P. salivae*, and the remaining 9 OTUs (6.5% of clones) showed sequence identities to several *Prevotella* species including *P. albensis*, *P. dentalis*, *P. ruminicola*, *P. multiformis*, *P. stercorea*, *P. bryantii* and *P. copri* (Table 2). Of the remaining 4 OTUs (of the 57 total OTUs), 2 OTUs (1.4% of clones) were distantly related (85%) to *Alistipes shahii*, 1 OTU (0.7% of clones) had 84% identity to *Barnesiella intestinihominis*, and 1 OTU (0.7% of clones) had 97% sequence identity to *S. dextrinosolvens*.

**Table 2:** Comparison of 16S rRNA gene libraries between the OL and CS groups

**OL group**					**CS group**				
**Phylotype**	**Clones**^**a**^	**OTU#**	**Nearest Taxon**	**%**^**b**^	**Phylotype**	**Clones**^**a**^	**OTU#**	**Nearest Taxon**	**%**^**b**^
SDMOL10	1	1	*P. brevis*	89	SDCS52	1	1	*P. brevis*	90
SDMOL20	1	2	*P. brevis*	89	SDCS61	1	2	*P. brevis*	90
SDMOL48	1	3	*P. brevis*	89	SDCS69	6	3	*P. brevis*	90
SDMOL69	1	4	*P. brevis*	89	SDCS71	2	4	*P. brevis*	90
SDMOL96	10	5	*P. brevis*	89	SDCS1	3	5	*P. brevis*	91
SDMOL29	2	6	*P. brevis*	89	SDCS74	1	6	*P. brevis*	91
SDMOL33	1	7	*P. brevis*	90	SDCS80	1	7	*P. brevis*	91
SDMOL38	1	8	*P. brevis*	90	SDCS14	1	8	*P. brevis*	92
SDMOL80	1	9	*P. brevis*	90	SDCS40	4	9	*P. brevis*	92
SDMOL91	1	10	*P. brevis*	90	SDCS49	3	10	*P. brevis*	92
SDMOL107	1	11	*P. brevis*	90	SDCS41	5	11	*P. brevis*	92
SDMOL108	1	12	*P. brevis*	90	SDCS5	1	12	*P. brevis*	93
SDMOL115	2	13	*P. brevis*	90	SDCS8	1	13	*P. brevis*	93
SDMOL120	2	14	*P. brevis*	90	SDCS93	6	14	*P. brevis*	93
SDMOL4	1	15	*P. brevis*	91	SDCS16	2	15	*P. brevis*	98
SDMOL27	2	16	*P. brevis*	91	SDCS85	1	16	*P. salivae*	90
SDMOL32	1	17	*P. brevis*	91	SDCS48	2	17	*P. salivae*	91
SDMOL84	2	18	*P. brevis*	91	SDCS2	1	18	*P. salivae*	92
SDMOL92	2	19	*P. brevis*	91	SDCS90	1	19	*P. ruminicola*	91
SDMOL17	5	20	*P. brevis*	92	SDCS98	5	20	*P. ruminicola*	92
SDMOL55	1	21	*P. brevis*	92	SDCS53	1	21	*P. ruminicola*	93
SDMOL68	8	22	*P. brevis*	92	SDCS54	3	22	*P. ruminicola*	93
SDMOL110	4	23	*P. brevis*	92	SDCS78	1	23	*P. ruminicola*	93
SDMOL70	1	24	*B. intestinihominis*	86	SDCS37	7	24	*P. ruminicola*	93
SDMOL5	1	25	*P. shahii*	86	SDCS44	1	25	*P. ruminicola*	94
SDMOL21	1	26	*P. shahii*	88	SDCS47	1	26	*P. ruminicola*	94
SDMOL71	2	27	*P. shahii*	89	SDCS94	1	27	*P. ruminicola*	94
SDMOL18	1	28	*P. shahii*	90	SDCS11	1	28	*P. ruminicola*	95
SDMOL30	1	29	*P. shahii*	90	SDCS9	5	29	*P. ruminicola*	95
SDMOL75	10	30	*P. shahii*	90	SDCS87	2	30	*Par. clara*	88
SDMOL76	1	31	*P. shahii*	90	SDCS7	1	31	*Par. clara*	89
SDMOL82	2	32	*P. shahii*	90	SDCS60	1	32	*P. shahii*	85
SDMOL88	1	33	*P. shahii*	90	SDCS76	2	33	*P. shahii*	85
SDMOL109	1	34	*P. shahii*	90	SDCS13	1	34	*P. shahii*	90
SDMOL118	1	35	*P. shahii*	90	SDCS86	1	35	*P. veroralis*	91
SDMOL7	1	36	*P. bryantii*	90	SDCS77	1	36	*P. veroralis*	92
SDMOL28	1	37	*P. copri*	87	SDCS104	1	37	*P. dentalis*	91
SDMOL26	1	38	*P. copri*	89	SDCS88	1	38	*P. albensis*	87
SDMOL135	1	39	*P. copri*	91	SDCS21	1	39	*Ros. hominis*	90
SDMOL34	1	40	*P. salivae*	89	SDCS28	1	40	*Pab. merdae*	84
SDMOL47	2	41	*P. salivae*	90	SDCS20	8	41	*S. dextrinosolvens*	97
SDMOL64	3	42	*P. salivae*	91	SDCS89	1	42	*Rum. bromii*	90
SDMOL74	3	43	*P. salivae*	91	SDCS36	1	43	*Rum. bromii*	95
SDMOL98	5	44	*P. salivae*	91	SDCS97	1	44	*Rum. bromii*	95
SDMOL139	3	45	*P. salivae*	92	SDCS38	1	45	*Pab. merdae*	84
SDMOL63	1	46	*P. veroralis*	91	SDCS50	1	46	*Pro. acetatigenes*	83
SDMOL44	16	47	*P. veroralis*	92	SDCS83	1	47	*A. shahii*	85
SDMOL136	16	48	*P. veroralis*	92	SDCS96	1	48	*Sp. acetigenes*	84
SDMOL53	1	49	*P. albensis*	91	SDCS102	1	49	*C. aldrichii*	86
SDMOL58	2	50	*P. stercorea*	87	SDCS105	1	50	*C. bolteae*	91
SDMOL100	1	51	*P. multiformis*	91					
SDMOL117	1	52	*P. ruminicola*	92					
SDMOL143	1	53	*P. dentalis*	91					
SDMOL31	1	54	*A. shahii*	85					
SDMOL37	1	55	*A. shahii*	88					
SDMOL127	1	56	*S. dextrinosolvens*	97					
SDMOL66	1	57	*P. brevis*	87					

*P Prevotella, S Succinivibrio, A Alistipes, Par Paraprevotella, Ros Roseburia, Rum Ruminococcus, Sp Sporanaerobacter, C Clostridium, Pab Parabacteroides, Pro Proteiniphilum, B Barnesiella*, *a *number of clones, *b *sequence indentity, *OTU # *OTU No.

Within the CS clone library, 36 of the 50 OTUs were 85-98% related to species belonging to genus *Prevotella*. Within these 36 OTUs, only one OTU (2% of clones) had >97% sequence identity to *P. brevis*, 14 OTUs (36% of clones) had 90-93% identity to *P. brevis* and 11 OTUs (27% of clones) had 91-95% identity to *P. ruminicola* making them the dominant bacterial species, whereas the remaining 10 OTUs (12% of clones) exhibited distant sequence identity to *P. shahii*, *P. veroralis*, *P. albensis*, *P. salivae* and *P. dentalis*. Of the remaining 14 OTUs (of the 50 total), 3 OTUs (3% of clones) were distantly related (89%) to *Paraprevotella clara*, 1 OTU (9% of clones) showed 97% identity to *S. dextrinosolvens*, 3 OTUs (3% of clones) had 90-95% identity to *Ruminococcus bromii*, 2 OTUs (2% of clones) had 84% identity to *Parabacteroides merdae*, 1 OTU (1% of clones) was 86% related to *Clostridium aldrichii*, and 1 OTU (1% of clones) was 91% related to *Clostridium bolteae*, 4 other OTUs (4% of clones) showed distant sequence identities to *Roseburia hominis*, *Proteiniphilum acetatigenes*, *A. shahii* and *Sporanaerobacter acetigenes*, respectively.

**Figure 3:**
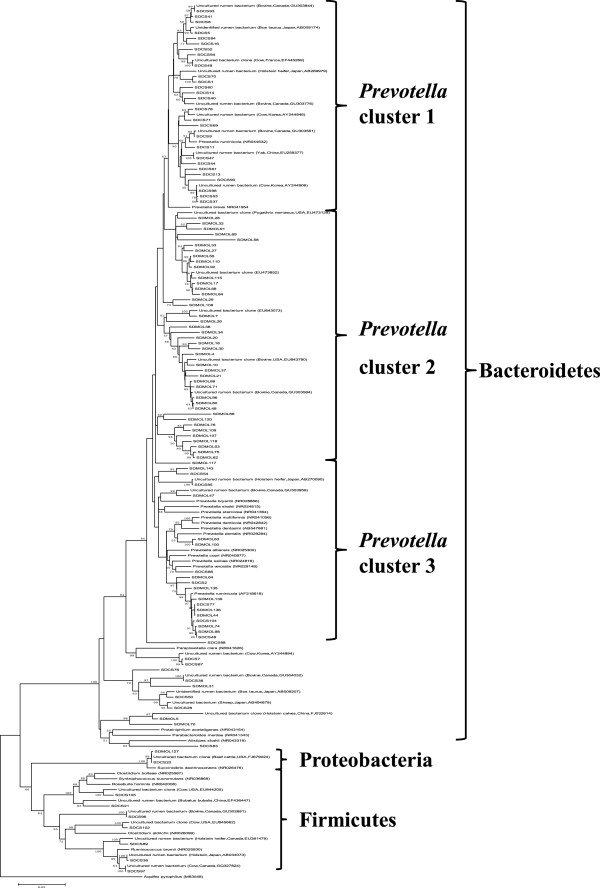
**Phylogenetic tree of bacterial 16S rRNA sequences from two groups using the Neighbor-Joining method and Kimura two-parameter model in MEGA. **Clones from Sika deer fed oak leaves beginning with SDMOL, followed by clone number, and from corn stalks beginning with SDCS, followed by clone number. *Aquifex pyrophilus* was used as the outgroup. Statistical significance was verified by bootstrapping 1000 replicates.

**Figure 4:**
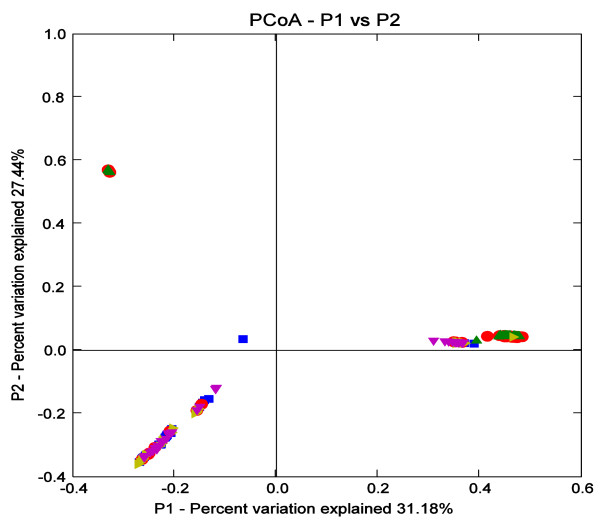
**PCoA analysis generating from the UniFrac software coloured by host animals and diets. **Norwegian reindeer fed concentrates were represented by dark-blue square; Norwegian reindeer consuming summer pasture were represented by yellow triangle; Svalbard reindeer consuming summer pasture were represented by purple triangle; domesicated Sika deer fed oak leaves were represented by green triangle and domesicated Sika deer fed corn stalks were represented by red round spot.

Overall, phylogenetic analysis revealed that the 107 OTUs were divided into six distinct phylogenetic groups (Figure 3).In addition, the comparison between Norwegian reindeer, Svalbard reindeer and domesticated Sika deer at community level with Fast Unifrac [13], which analyze phylogenetic lineages, showed that the bacterial composition in the rumen of domesticated Sika deer fed oak leaves based diets was more similar to that of domesti-cated Sika deer fed corn stalks based diets, and differed from Svalbard reindeer and Norwegian reindeer (Figure 4). However, there were also shared bacterial communities between domestic Sika deer and Reindeer.

### Rumen bacterial diversity based on the PCR-DGGE profile

**Figure 5:**
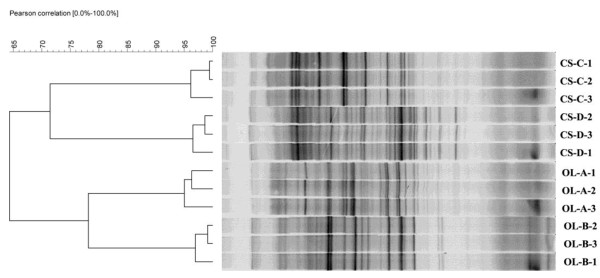
**PCR-DGGE profiles of the rumen bacterial 16S rNA gene (V3 region) from domestic Sika deer fed oak leaves (Sika deer A and B) and corn stalks (Sika deer C and D). **OL and CS represented Sika deer fed oak leaves and corn stalks, respectively. Three replicates (1, 2 and 3) were taken from each Sika deer. Bionumerics software generated the clustering dendrogram using the UPGMA method.

PCR-DGGE banding profiles showed that the bacterial communities clustered with respect to diets (Figure 5). However, considerable animal-to-animal variation was also observed. A distinct difference in the bacterial structure was observed between two diets. By comparing the PCR-DGGE profiles between the two diets, the number of DGGE bands from CS group was considerably abundant compared to those from OL group (Figure 5). There were also several bands that were common for all domestic Sika deer.

In total, 47 dominant bands were excised from the PCR-DGGE profile and sequenced, of which 20 and 27 bands obtained from the OL and CS groups, respectively (see Additional file 1). Sequences from the excised bands from the OL group belonged to the phyla Firmicutes, Bacteroidetes and Proteobacteria, whereas DGGE sequences from the CS group belonged to the phyla Firmicutes, Bacteroidetes, Proteobacteria and Synergistetes. Among the 47 bands, 13 bands in two groups were identified as known species based on ≥ 97% sequence similarity (Table 3). Bands O-1, C-3 and C-5 showed ≥ 98% similarity with known species of *C. populeti* 743A. Bands O-3 and O-18 were identified as *Streptococcus pasteurianus* CIP 107122, while bands O-9 and C-14 showed 98% similarity with of *Eubacterium cellulosolvens* 6. Band O-12 displayed 97% similarity with known species of *Moryella indoligenes* AIP 220.04, and band O-13 showed species-level sequence similarity to *Pseudobutyrivibrio ruminis* DSM9787. Bands O-10 and C-10 displayed 98% similarity to *Succinivibrio dextrinosolvens* 0554, while bands C-18 and C-1 had 98% sequence similarity to *Coprococcus eutactus* ATCC 27759 and *Prevotella ruminicola* ATCC 19189, respectively. Moreover, band C-21 had the 93% similarity with known species of *Eubacterium ruminan-tium* GA 195. Bands C-13 and C-22 were distantly related to *Galbibacter mesophilus* Mok-17 with 88% and 91% similarity, respectively. Band C-24 displayed 88% similarity with *Capnocytophaga cynodegmi* CIP 103937, and band C-27 showed 94% similarity with known species of *Bacteroides uniformis* JCM 5828. Bands C-19 and C-20 had 92% similarity with known species of *Dethiosulfovibrio acidaminovorans* sr15. The remaining 30 bands from two groups had 92-96% sequence similarities with several species belonging to genus *Prevotella* including *P. loescheii*, *P. pleuritidis*, *P. corporis*, *P. buccalis*, *P. dentalis*, *P. melani-nogenica*, *P. salivae*, *P. copri*, *P. denticola*, *P. oulorum* and *P. histicola*.

**Table 3:** Sequences analysis of V3 region of 16S rRNA gene from PCR-DGGE

**OL group**			**CS group**		
**Band No**	**Nearest cultured relative (GenBank accession No)**	**%**^**a**^	**Band No**	**Nearest cultured relative (GenBank accession No)**	**%**^**a**^
O-1	*C. populeti* (NR026103)	99	C-1	*P. ruminicola* (NR044632)	98
O-2	*P. salivae* (NR024816)	93	C-2	*P. loescheii* (NR043216)	96
O-3	*St. pasteurianus* (NR043660)	100	C-3	*C. populeti* (NR026103)	98
O-4	*P. dentalis* (NR029284)	94	C-4	*P. pleuritidis* (NR041541)	94
O-5	*P. salivae* (NR024816)	96	C-5	*C. populeti* (NR026103)	98
O-6	*P. denticola* (NR042842)	95	C-6	*P. pleuritidis* (NR041541)	94
O-7	*P. oulorum* (NR029147)	94	C-7	*P. corporis* (NR044627)	94
O-8	*P. buccalis* (NR044630)	94	C-8	*P. buccalis* (NR044630)	94
O-9	*E. cellulosolvens* (NR026106)	98	C-9	*P. dentalis* (NR029284)	95
O-10	*S. dextrinosolvens* (NR026476)	98	C-10	*S. dextrinosolvens* (NR026476)	98
O-11	*P. salivae* (NR024816)	95	C-11	*P. dentalis* (NR029284)	93
O-12	*M. indoligenes* (NR043775)	97	C-12	*P. melaninogenica* (NR042843)	95
O-13	*Ps. ruminis* (NR026315)	99	C-13	*G. mesophilus* (NR041450)	88
O-14	*P. oulorum* (NR029147)	94	C-14	*E. cellulosolvens* (NR026106)	98
O-15	*P. dentalis* (NR029284)	94	C-15	*P. dentalis* (NR029284)	95
O-16	*P. histicola* (NR044407)	95	C-16	*P. loescheii* (NR043216)	93
O-17	*P. dentalis* (NR029284)	95	C-17	*P. salivae* (NR024816)	88
O-18	*St. pasteurianus* (NR043660)	100	C-18	*Cp. utactus* (NR044049)	98
O-19	*P. dentalis* (NR029284)	96	C-19	*D. acidaminovorans* (NR029034)	92
O-20	*P. dentalis* (NR029284)	96	C-20	*D. acidaminovorans* (NR029034)	92
			C-21	*E. ruminantium* (NR024661)	93
			C-22	*G. esophilus* (NR041450)	91
			C-23	*P. copri* (NR040877)	92
			C-24	*Ca. cynodegmi* (NR043063)	88
			C-25	*P. copri* (NR040877)	93
			C-26	*P. dentalis* (NR029284)	94
			C-27	*B. uniformis* (NR040866)	94

*C Clostridium, E Eubacterium, P Prevotella, S Succinivibrio, St Streptococcus, M Moryella, Ps Pseudobutyrivibrio, Cp Coprococcus, G Galbibacter, Ca Capnocytophaga, B Bacteroides, D Dethiosulfovibrio*, *a* sequence similarity.

## Discussion

In the present study, two 16S rRNA gene libraries and PCR-DGGE were used to study the rumen bacteria in the rumen of domesticated Sika deer feeding on oak leaves-based (OL) and corn stalks-based (CS) diets. Sequences from the two clone libraries and PCR-DGGE bands indicated that the majority of sequences belonged to phylum Bacteroidetes. The findings from the current study are similar to previous findings for other ruminants, such as Reindeer, yaks, cattle and goats [14-18]. The predominance of sequences belonging to the phylum Bacteroidetes highlights their important role in the rumen fermentation of domesticated Sika deer. While, the phylum Firmicutes being prevalent in other ruminants were not found in the OL library [19], which could be caused by the tannins contained in oak leaves, because some studies reported that the tannins in oak leaves may have a negative effect on some of the bacterial species [20,21], and the growth of proteolytic bacteria, such as *Butyrivibrio fibrisolvens*, *Ruminococcus albus* and *Streptococcus bovis*, were inhibited by tannins [22,23]. This may also indicate that some species belonging to phylum Firmicutes in the rumen of domestic Sika deer may be sensitive to tannins.

Within the phylum Bacteroidetes, *Prevotella*-like clones accounted for 97.2% of the clones in the OL group and 77% in the CS group. Moreover, the PCR-DGGE results also showed the genus *Prevotella* represented the predominant bacteria in rumen of domesticated Sika deer (Table 3), which is in agreement with other studies [19,24-28] . The prevalence of *Prevotella* spp. in rumen fermentation of domesticated Sika deer was likely because they utilize a wide variety of polysaccharides, and are thought to be important contributors to xylan degradation in the rumen [29-32]. Although other studies found that concentrate diets increased the numbers of clones related to *Prevotella* spp. [33,34], however, in comparison with other ruminants, there was an apparent difference in the proportion of *Prevotella* spp. [6,25,27,28]. *Prevotella* spp. belonged to the hydrogen-consuming bacteria, which could produce propionate via succinate or acrylate pathways though fermentation of sugars and lactate, respectively [35-37]. Therefore, the dominant genus *Prevotella* in the rumen of domesticated Sika deer suggested that the propionate pathway may be relatively vital in the rumen fermentation of domestic Sika deer, which, in turn, may lead to the decreased production of methane, since the succinate-propionate pathway could compete with methanogens for hydrogen [38]. The relationship between *Prevotella* spp. and methanogens in the rumen of domesticated Sika deer was worth of further investigating. In addition, the bacterial communities in the rumen between domesticated Sika deer, Svalbard reindeer and Norwegian reindeer, all cervids, were compared using Fast UniFrac, which can be used to determine whether communities are significantly different [13]. The results of Principal coordinate analysis (PCoA) between domesticated Sika deer and Reindeer using the Fast Unifrac platform clearly showed that the rumen bacterial communities were distinct, which can be attributed to the host-species (Figure 5) [13,26,39].

It is important to note, that fibrolytic bacteria, such as *C. populeti*, *E. cellulosolvens* and *Ps. ruminis* were discovered in our analysis based on PCR-DGGE, rather than the predominant fibrolytic bacteria, *B. fibrisolvens*, *Fibrobacter succinogenes*, *Ruminococcus flavefaciens* and *R. albus*. This may suggest that the rumen of domesticated Sika deer depend on unique bacterial communities in rumen fermentation. In contrast, the absence of *R. flavefaciens*, *B. fibrisolvens*, *F. succinogenes* and *R. albus* in the present work may be attributed to the small number of clones may have missed some other members of the bacterial community, and the weak or unidentifiable bands in DGGE. Future work will employ next generation sequencing to effectively elucidate the bacterial diversity present in the rumen of domesticated Sika deer and other livestock. Collectively, these data indicated that the rumen of domesticated Sika deer harbored unique bacterial populations for the fermentation of plant biomass and concentrate diet.

Interestingly, in both clone libraries, none of the sequences were 100% identical. Rather, most clones were in the range of 83-98% identify to known species in both libraries. These results suggested that the rumen bacteria of domesticated Sika deer were not previously characterized and that these clones related to *Prevotella* spp. in the rumen represented new species. This agrees with previous findings suggesting that most of the bacterial species in rumen of other cervids (96% for Hokkaido Sika deer and 100% for Svalbard reindeer) are unknown [26,40]. Despite the diets and geographic location are important factors affecting bacterial diversity in the rumen, however, the presence of these unknown or unidentified species may be the result of co-evolution between microbial communities and the host.

PCR-DGGE analysis showed that the bacterial diversity in domesticated Sika deer fed corn stalks differed from the domesticated Sika deer consuming oak leaves (Figure 5), indicating forage affected the relative abundance and composition of the bacteria. Moreover, the difference in the *Prevotella* species between the two groups was very apparent (Table 3). For instance, the results of clone library showed that the proportion of *P. ruminicola*-like clones (27%) was abundant in the CS group comparing with those in the OL group, and sequences analysis of PCR-DGGE also indicated that *P. ruminicola* was only presented in CS group. Interestingly, *Prevotella* species in the rumen could contribute to cell wall degradation through synergistic interactions with species of cellulolytic bacteria [41]. Therefore, considering the relatively high fiber content (about 36%) in corn stalks, these *P. ruminicola*-like clones in the CS group may play a role in the degradation of cellulose. This explanation is partly supported by recent metagenomics data from the Svalbard reindeer rumen microbiome, where the presence of polysaccharide utilizing glycoside hydrolase and other carbohydrate-active enzyme families target various polysaccharides including cellulose, xylan and pectin [18].

In the OL group, the distribution of *P. shahii*-like clones (16.5%), *P. veroralis*-like clones (23.8%) and *P. salivae*-like clones (12.3%) were several times higher in the OL library than in the CS library, and several bands in the PCR-DGGE analysis showed sequence similarities to *P. salivae* (Table 3). Previous study reported that *P. ruminicola* may tolerate condensed tannins [22]. Considering the genetic diversity of *Prevotella* spp. [27,42], it is assumed that the tolerance to tannins of domestic Sika deer may be related to the abundance of *Prevotella* spp. in the OL group. In addition, we found two bands (O-3 and O-18) were identified as *St. pasteurianus* using PCR-DGGE. Thus this species may also be important in the process of degrading tannins in diets, because tannin-degrading capability of *Streptococcus* sp. have been demonstrated in other studies [43-46]. However, these assumptions need to be investigated in future studies.

Phylogenetic analysis indicated the presence of diet-specific subpopulations of *Prevotella*. *Prevotella* clusters 1 and 2 not only demonstrated the genetic diversity of *Prevotella* spp., but also confirmed the above assumption that clones grouped within clusters 1 or 2 may be related to the degradation of fiber (cluster 1) or tannins (cluster 2), whereas, the clones in cluster 3 may have common features of degrading starch and proteins contained in concentrate diets (Figure 3). However, clones related to the bacterial genera *Sporanaerobacter*, *Parabacteroides* and *Proteiniphilum* were found in the rumen of domesticated Sika deer fed corn stalks that were not previously reported in the rumen from other ruminants. *Sporanaerobacter acetigenes* is an acetogenic and a sulfur-reducing bacterium that was isolated from an anaerobic sludge blanket reactor in Mexico [47,48]. The rumen has considerable capacity to convert sulfate into sulfur-containing amino acids. Similarly, little is known about *Proteiniphilum acetatigenes*, which was originally isolated from a UASB reactor treating brewery wastewater in China [49]. These bacteria in rumen of domesticated Sika deer may have other biological functions and is worthy of further investigation.

## Conclusions

In conclusion, this study is the first to report the rumen bacteria in Chinese domesticated Sika deer, consuming either oak leaves-based or corn stalks-based diets. Sequences analysis from 16S rRNA clone libraries and PCR-DGGE revealed that the domesticated Sika deer harbored unique rumen bacterial populations, most of which may present novel species, and that the bacterial compositions were affected by forage. It is speculated that the possible new species of *Prevotella* may be related to the degradation of tannins or fiber biomass. Moreover, the species diversity of *Prevotella* sp. in the rumen combined with their synergistic interactions with other microorganisms requires further in depth investigation.

## Methods

### Animals and sampling

Four male rumen-cannulated domestic Sika deer (*Cervus nippon*) maintained at the research farm (44.04° N, 129.09° E) of the Institute of Special Animal and Plant Sciences, Chinese Academy of Agricultural Sciences, in Jilin Province, were used in this study. From September to October, four domestic Sika deer were offered the same concentrated diets (64.5% corn, 19.7% soybean meal, 12.8% distiller dried grains with solubles and a 3% mixture of vitamins and mineral salts) and mixed with either oak leaves (OL) or corn stalks (CS). All domestic Sika deer were fed twice each day at 8:00 AM and 4:00 PM and had free access to water. The whole rumen contents, which included solid and fluid fractions, were collected via rumen cannula before the morning feeding, and stored at -20°C for analysis. All domestic Sika deer used in present experiment must be performed according to the animal health and well-being regulations, all animal procedures were approved and authorized by the Chinese Academy of Agricultural Sciences Animal Care and Use Committee, and by the Wild Animal and Plant Subcommittee, Institute of Special Animal and Plant Sciences.

### DNA extraction

Total DNA was directly extracted from rumen contents containing solid and liquid fraction according to methods described by LaMontagne [50] with few modifications. In brief, 800 μl lysis buffer (0.15 M NaCl, 0.2 M EDTA, 10 mg.ml^-1^ lysozyme, pH8.0), 20 μl of 20 mg.ml^-1^ proteinase K (Sigma, Germany), and 0.3 g glass beads (0.1 mm, Sigma, Germany) were added to 0.5 g of whole rumen contents. After shaking at 37°C for 1 h, 300 μl heated lysis buffer (10% SDS, 0.1 M NaCl, 0.5 M Tris–HCl, pH8.0) at 65°C, 300 μl phosphate buffer (pH8.0) and 600 μl chloroform-isoamyl alcohol (24:1, V/V) were added, and the mixture was incubated at 65°C in a water bath for 30 min with intense shaking 30 s at 10 min intervals. After centrifugation at 5,000 rpm for 6 min, the supernatant was transferred to a clean tube. DNA was then precipitated with a 0.6 volume of isopropanol at -80°C for 15 min, and the pellet was washed several times with 75% ethanol. The DNA was dried and dissolved in TE buffer (pH 8.0). The DNA quality was assessed by 0.8% agarose gel electrophoresis, and the purity was determined by spectrophotometry (SPECORD 50, analytikjena, Germany), after which it was purified using a QIAEX II Gel Extraction Kit (QIAGEN, Germany).

### Construction of 16S rRNA gene clone libraries and sequences analyses

Universal primers 27F (5^′^-AGAGTTTGATCMTGGCTCAG-3^′^) and 1492R (5^′^-TACGGYTACCTTGTTACGACTT-3^′^) were used to amplify the 16S rRNA gene (approximately 1.5 kb) [51]. Each 50 ul reaction contained 50 ng template DNA, 0.25 mM of each primer, 250 mM dNTPs, 1.25 U of Ex Taq and 5 μl Ex Taq buffer (TaKaRa, Dalian). PCR was performed on a 2720 Thermal Cycler (Applied Biosystems, USA) with hot start at 94°C for 5 min, followed by 20 cycles of 30 s at 94°C, 1 min at 55°C and 2 min at 72°C; and a final extension at 72°C for 10 min. The PCR product was assessed using 2% agarose gel electrophoresis (approximately 1.5 kb), and were purified using a TaKaRa MiniBEST DNA Fragment Purification Kit (TaKaRa, Dalian) and then pooled within each group. Two 16S rRNA gene clone libraries were constructed from the pooled PCR products using the TOPO^®^ TA Cloning^®^ Kit (Invitrogen, USA). Positive (white) clones were screened by colony PCR with the M13 Forward and M13 Reverse primers, and sequenced using an ABI 3730XL DNA Analyzer.

The chimera check program Bellerophon was used to identify chimeric sequences [52]. The remaining sequences were assigned using the Classifier tool available at Ribosomal Database Project (RDP) Release 10 at a confidence threshold of 80% [53]. OTUs based on 97% sequence identity, and the Shannon-Wiener index-based diversity estimator and the Chao1 based index of richness were calculated using MOTHUR platform to determine the diversity and richness of bacterial communities in each group based on the 16S rRNA gene libraries [54]. Libshuff analysis was performed to estimate the similarity between libraries from two diets based on evolutionary distance of all sequences. Coverage and rarefaction curves were also determined using the MOTHUR platform [54]. The 16S rRNA gene sequences were screened using GenBank’s BLAST program [55]. The closest related sequences were retrieved and aligned with sequences from the present study using the CLUSTALW 1.83 program in MEGA 5.05 software [56]. A phylogenetic tree was constructed using the Kimura two-parameter model and the Neighbor-Joining method as part of the MEGA 5.05 software. The statistical significance was verified by 1000 bootstrapped replicates. The sequences obtained from this study were submitted to GenBank under the accession numbers JX889268 to JX889378. Furthermore, an unweighted UniFrac distance matrix was constructed from the phylogenetic tree of clone libraries of Norwegian reindeer, Svalbard reindeer and Sika deer, and was visualized using PCoA [13,26,39].

### PCR-DGGE banding profiles and statistical analysis

The variable region (V3) of the bacterial 16S rRNA gene was amplified using the primers of F341GC and R534, and PCR condition was described previously [57]. A 40 bp GC-clamp (5′-CGCCCGGGGCGCGCCCCGGGCGGGGCGGGGGCACGGGGGG-3′) was on the 5′ end of the F341 primer. The PCR products were loaded onto 8% polyacrylamide gels (37.5:1) with a denaturing gradient of 40–60% at 80V over 16 h at 60°C. Electrophoresis was performed using Bio-Rad’s DCode detection system. The gels were stained with SYBR Green I (Invitrogen, USA) for 25 min and gel images were captured using the Gel Doc™ XR^+^ system (BIO-RAD, CA). Cluster analysis was performed using a Dice similarity coefficient at 0.5% optimization and 1% tolerance following the unweighted pair-group method using arithmetic averages (UPGMA) on BioNumerics 6.0 software (Applied-Maths, Kortrijk, Belgium).

Dominant bands were excised from DGGE gel and eluted overnight in 500 μl of sterilized ddH_2_O at 4°C. Extracted DNA was re-amplified using PCR primers F341 and R534 without GC-clamp. The size of PCR products were determined using agarose gel and were purified using QIAquick^®^ PCR Purification Kit (Qiagen, USA). The PCR products were cloned into TOPO^®^ TA Cloning^®^ Kit with TOP 10 according to the manufacturer’s instruction (Invitrogen, San Diego, CA, USA). Recombinant plasmids of positive clones (white) were sequenced using ABI 3730XL DNA Analyzer. The sequences were compared with those sequences deposited in NCBI web site using BLAST program [55].

## Abbreviations

DGGE: Denaturing gradient gel electrophoresis; OTUs: Operational taxonomic units; VFA: Volatile fatty acids; BLAST: Basic local alignment search tool; RDP: Ribosomal database project; PCoA: Principal coordinates analysis.

## Competing interests

The authors declare that they have no competing interests.

## Authors’ contributions

ZPL sampled rumen contents, extracted DNA, constructed the clone library, data analysis and drafted the manuscript. ADGW was involved with interpretation of data and with preparing the manuscript. HLL designed the study and drafted the paper. KB, YFY, CX and KYW contributed to sample rumen contents and all of lab works. GYL and FHYconceived the study. All authors read and approved the final manuscript.

## Supplementary Material

Additional file 1 **Dominant bands of PCR-DGGE banding patterns of bacteria 16SrRNA gene (V3 region).** In the text, bands from OL group were defined as O and followed by bands number, bands from CS group begin with C and followed by bands numbers. Click here for file
