# Supplementary figures and images for: Molecular diversity of rumen bacterial communities from tannin-rich and fiber-rich forage fed domestic Sika deer (Cervus nippon) in China
Source: BMC Microbiol. 2013 Jul 8;13:151. doi: 10.1186/1471-2180-13-151 (PMC3723558; doi:10.1186/1471-2180-13-151)

A-1 A-2 A-3 B-1 B-2 B-3 C-1 C-2 C-3 D-1 D-2 D-3

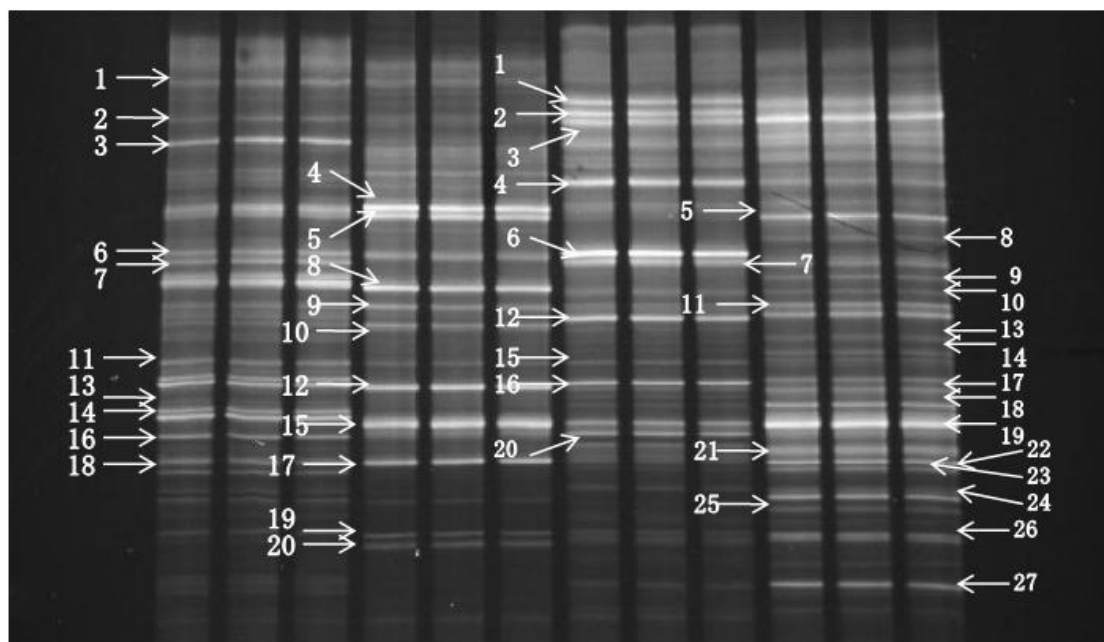

Supplement: Additional file 1 — Dominant bands of PCR-DGGE banding patterns of bacteria 16SrRNA gene (V3 region). In the text, bands from OL group were defined as O and followed by bands number, bands from CS group begin with C and followed by bands numbers. [file 1471-2180-13-151-S1.pdf]
